# Acute Fulminant Myocarditis Secondary to Coxsackie B Virus

**DOI:** 10.7759/cureus.79635

**Published:** 2025-02-25

**Authors:** Suhas R Seshadri, Edward Salem, Andrew Carlson, Keshav Patel, Marco Shaker

**Affiliations:** 1 Internal Medicine, University of Illinois at Chicago, Chicago, USA; 2 Cardiology, University of Illinois at Chicago, Chicago, USA

**Keywords:** coxsackie b virus, fulminant myocarditis, mri cardiac, myocarditis, short term mechanical circulatory support

## Abstract

Fulminant myocarditis is a severe form of acute myocarditis, characterized by complications such as hemodynamic compromise, often requiring advanced mechanical support. It can occur as a result of infection, toxin exposure, or autoimmune processes. This is a case of a 53-year-old male with no past medical history who presented with one week of fever, shortness of breath, and upper respiratory symptoms and subsequently developed fulminant myocarditis, characterized by severe left ventricular dysfunction and cardiogenic shock requiring both inotropic and advanced mechanical support. Initial serological diagnostics were negative for a viral etiology; however, repeat testing, prompted by continued high clinical suspicion, subsequently yielded a positive *Coxsackie B* viral titer. The diagnosis of myocarditis was further supported by the specific distribution of late gadolinium enhancement (LGE) on cardiac MRI. We describe the nuanced management approach, including heart failure guideline-directed medical therapy (GDMT), high-dose steroids, and intravenous immunoglobulins. Additionally, we highlight the role of noninvasive diagnostics and the challenges in targeted management of fulminant myocarditis.

## Introduction

Myocarditis is an inflammatory disease of the myocardium that can occur as a result of infection, toxin exposure, or immune system activation. It typically presents with chest pain and shortness of breath and most commonly follows an uncomplicated, self-limited clinical course [1]. In severe cases, patients may develop cardiogenic shock, ventricular arrhythmias, atrioventricular blocks, or sudden death [1]. The incidence of myocarditis has increased with the use of cardiac MRI, with the number of reported cases per 1 million people in the United States rising from 95 in 2005 to 114 in 2014 [2]. During that period, the overall in-hospital mortality for myocarditis patients was 4.43% [2].

While endomyocardial biopsy remains the gold standard for diagnosing myocarditis, particularly for histological subtyping, it is pursued selectively in high-risk presentations. A noninvasive workup can establish the diagnosis of "clinically suspected myocarditis" [1]. This workup may include laboratory testing with inflammatory markers, viral studies, and high-sensitivity troponin, as well as ECG and rhythm monitoring, stress testing, and cardiac imaging, including echocardiography, catheterization, and cardiac MRI. Myocarditis treatment generally consists of supportive management, treatment of heart failure in patients with this presentation, and selective use of immunosuppressive agents as indicated.

Fulminant myocarditis is a severe form of acute myocarditis characterized by hemodynamic compromise requiring advanced hemodynamic support measures, such as inotropic agents, Impella devices, intra-aortic balloon pumps (IABP), and extracorporeal membrane oxygenation (ECMO). Management in very severe cases may necessitate heart transplant evaluation [3]. Complications of fulminant myocarditis include ventricular arrhythmias, multiorgan failure, and death, making early diagnosis and treatment essential. Different imaging modalities provide specific diagnostic information. On transthoracic echocardiogram, fulminant myocarditis presents with severe left ventricular (LV) dysfunction and wall motion abnormalities that do not always correspond to coronary artery distributions. Additional findings may include pericardial effusions, right ventricular dysfunction, and diffuse wall thickening. Cardiac MRI (CMR) is particularly useful in identifying myocarditis, as late gadolinium enhancement (LGE) typically appears in a nonischemic pattern-subepicardial (midwall) or intramural zones dispersed throughout the ventricular walls [3]. It is estimated that 5% to 10% of patients admitted with acute myocarditis can be classified as having fulminant myocarditis [3]. We present a rare case of fulminant myocarditis that encompassed the above features and required multiple diagnostics to determine its viral etiology.

## Case presentation

**Figure 1:**
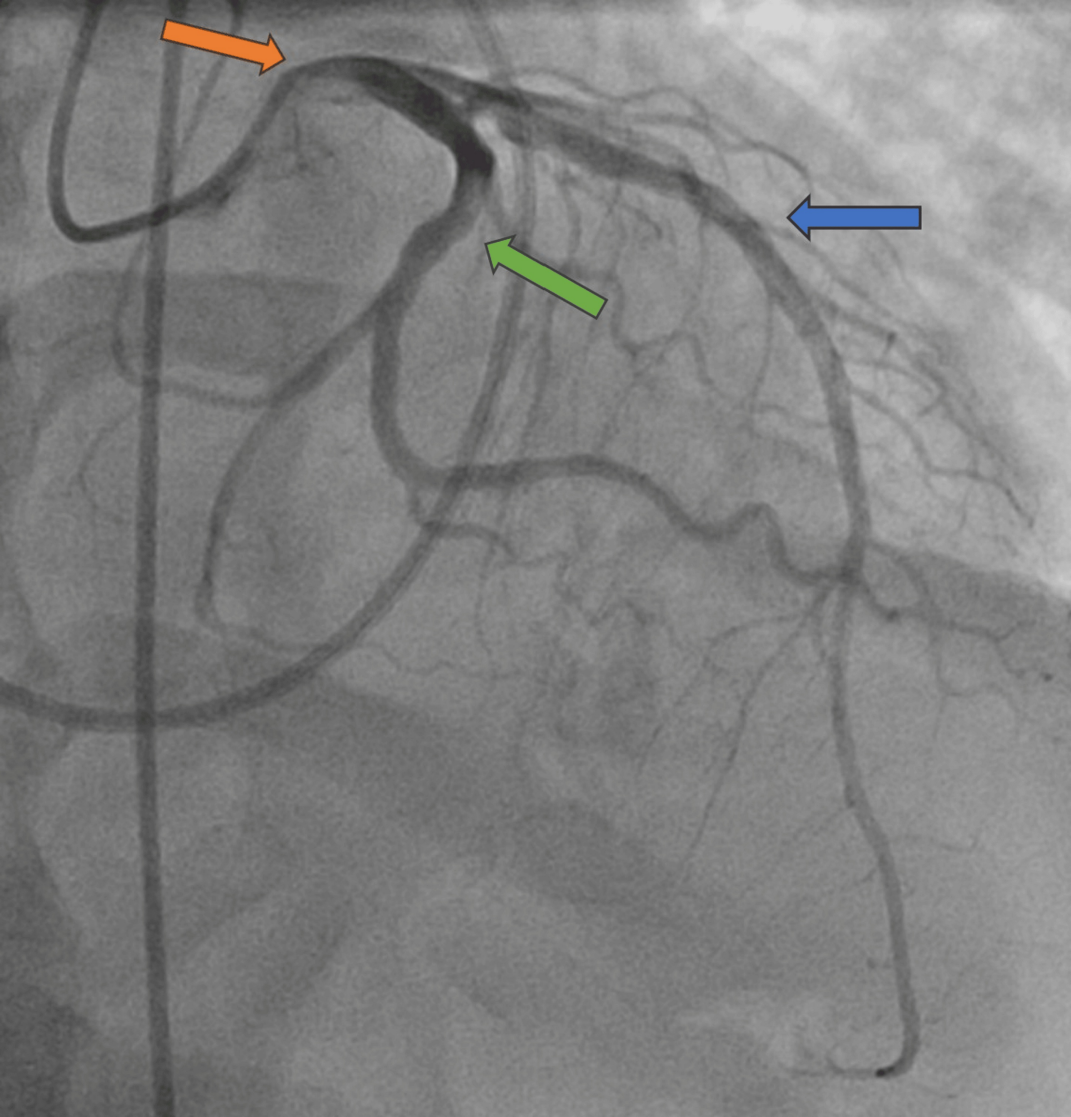
Coronary angiogram revealing patent vasculature in the left main stem (orange arrow), left anterior descending (blue arrow), and left circumflex (green arrow) arteries.

**Figure 2:**
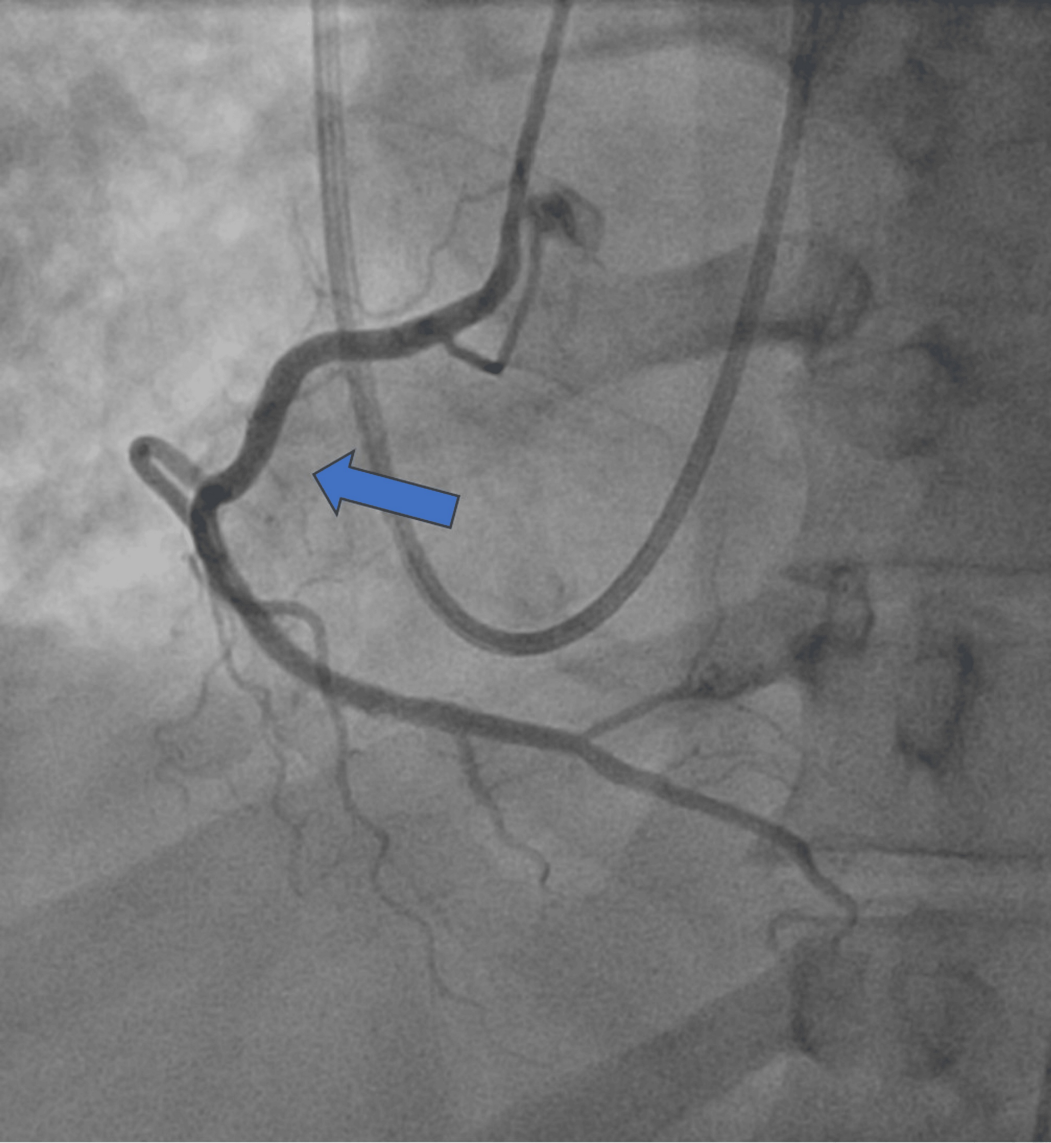
Coronary angiogram revealing patent right coronary artery (blue arrow).

A 53-year-old male with no past medical history presented with one week of cough, shortness of breath, myalgias, and fever. He was found to be tachycardic, with a heart rate of 133 beats per minute; hypotensive, with a blood pressure of 85/65 mmHg; and hypoxic, with an oxygen saturation of 88% on room air. Initial workup revealed an elevated high-sensitivity troponin level of 58,000 ng/L. ECG showed sinus tachycardia with ST depressions in the inferior and lateral leads. While myocarditis was considered given the presenting symptoms, Type 1 myocardial infarction needed to be ruled out. The patient was started on a heparin drip and received an antiplatelet loading dose. Coronary angiography revealed normal coronary arteries (Figures 1, 2); however, right heart catheterization demonstrated biventricular overload with elevated right and LV end-diastolic pressures of 12 mmHg and 35 mmHg, respectively.

The Fick cardiac output and cardiac index were decreased at 3.1 L/min and 1.58 L/min/m², respectively. An Impella heart pump was inserted, and milrinone was started for the management of cardiogenic shock. Transthoracic echocardiography confirmed a newly reduced left ventricular function (LVEF) of <20% with diffuse LV dysfunction without specific wall motion abnormalities, suggesting a nonischemic etiology. The workup at this time excluded Type 1 myocardial infarction, opening an exploration of Type 2 myocardial infarction etiologies with a working diagnosis of myocardial infarction with nonobstructive coronary arteries (MINOCA). Additionally, the hemodynamic studies in the right heart catheterization and the need for mechanical circulatory support with the Impella heart pump highlighted the severity of the cardiogenic shock this patient presented with.

Further workup revealed an elevated BNP (brain natriuretic peptide) of 726 pg/mL, LDH (lactate dehydrogenase) of 842 U/L, WBC (white blood cell) of 12.4 K/µL (neutrophils:lymphocytes, 83:10%), ESR (erythrocyte sedimentation rate) of 89 mm/h, and CRP (C-reactive protein) of 219 mg/L. An initial respiratory viral panel was negative. Given the inflammatory markers and the overall presenting clinical story, fulminant myocarditis was the presumed diagnosis. The rheumatology and infectious disease teams were consulted due to concern for infectious and immune-mediated myocarditis. With his deteriorating clinical picture, empiric treatment outweighed the risks. He was started on IVIG at 2 g/kg and pulse-dose steroids with IV methylprednisolone 500 mg twice daily empirically for immune-mediated fulminant myocarditis. Broad-spectrum antibiotics for concomitant community-acquired pneumonia were initiated; however, infectious workup for bacterial and fungal etiologies via culture data was later negative.

As his hemodynamics worsened, the patient was transferred to an outside hospital for possible extracorporeal membrane oxygenation (ECMO) or other advanced heart failure support, which he did not end up requiring. Endomyocardial biopsy, which would have allowed for targeted therapy from histological subtyping, was deferred due to significant hemodynamic instability. Due to high purge pressures associated with hemolysis and clotting, his Impella pump was exchanged for an IABP after transfer. His IVIG (intravenous immunoglobulin) course (total of four doses) was completed, and he was continued on daily oral steroids. He was managed with diuresis and heart failure guideline-directed medical therapy (GDMT), specifically metoprolol succinate and torsemide. Other therapies were not initiated in the setting of hypotension. His clinical condition improved, with milrinone successfully weaned off. Eventually, his IABP was removed. He was discharged on an extended oral steroid taper as well as metoprolol succinate and torsemide for his heart failure, with plans for outpatient follow-up for further optimization.

The etiology of his fulminant myocarditis was further investigated (Table 1). Initial infectious workup, including a Coxsackie B viral titer prior to transfer, was negative. Rheumatologic workup, including systemic lupus erythematosus, Sjögren’s syndrome, mixed connective tissue disease, rheumatoid arthritis, and myositis, was negative. He did have a positive PR3 antibody, which questioned the etiology of his myocarditis; however, there was no clinical evidence of ANCA vasculitis. After discussion, this antibody elevation was thought to be due to viral infection. Given the vast differential for etiologies, the patient was treated broadly as described above. With continued clinical suspicion and transfer to a new facility, repeat viral workup was performed, revealing elevated Coxsackie B (1:160) and echovirus (1:160) antibody titers. These repeated tests confirmed the diagnosis of a recent viral infection.

**Table 1:** Comprehensive infectious and rheumatologic workup for etiologies of fulminant myocarditis seen in this case. The infectious workup included a standard respiratory viral panel, Coxsackie viral titers, tuberculosis testing, and fungal testing for histoplasmosis and blastomycosis, as well as atypical bacterial etiologies such as Mycoplasma, *Chlamydia pneumoniae*, and *Bordetella pertussis*. Negative Coxsackie and echovirus viral titers prior to transfer (*) were compared to positive titers on repeat testing after transfer to OSH (**). The rheumatologic workup included systemic lupus erythematosus, Sjögren’s syndrome, mixed connective tissue disorder, rheumatoid arthritis, and myositis. Negative findings ruled out underlying autoimmune processes. An elevated PR3 antibody was deemed to be secondary to viral infection rather than vasculitis process. SARS-CoV-2: severe acute respiratory syndrome coronavirus 2, Ab: antibody, ANCA: antineutrophil cytoplasmic antibody, IFA: immunofluorescence assay, dsDNA: double-stranded deoxyribonucleic acid, ENA: extractable nuclear antigen, IgG: immunoglobulin G, IgA: immunoglobulin A, IgM: immunoglobulin M, PR-3: serine protease 3, AU: arbitrary units.

Test Name	Result
Infectious workup	
Adenovirus	Negative
Coronavirus (229E, HKU1, NL63, OC43)	Negative
SARS-CoV2	Negative
Human metapneumovirus	Negative
Human rhinovirus/enterovirus	Negative
Influenza A (H1-2009, H1, H3)	Negative
Influenza B	Negative
Parainfluenza virus 1-4	Negative
Respiratory syncytial virus	Negative
Bordetella pertussis	Negative
Mycoplasma pneumonia	Negative
Chlamydia pneumoniae	Negative
Coxsackie B virus Ab types 1-6*	Negative; (normal <1:10)
Coxsackie A virus serotype titer*	Negative; (normal <1:10)
QuantiFERON tuberculosis antigen	Negative
Histoplasmosis urine antigen	Negative
Blastomycosis urine antigen	Negative
Coxsackie B virus Ab titer**	Positive; 1:160 (normal <1:10)
Echovirus Ab titer**	Positive; 1:160 (normal <1:10)
Rheumatologic workup	
ANCA IFA titer	Negative; <1:20
Anti-dsDNA Ab	Negative
SSA-52 (Ro52) (ENA) antibody, IgG	Negative
SSA-60 (Ro60) (ENA) antibody, IgG	Negative
SSB (La) (ENA) antibody, IgG	Negative
SMITH/RNP (ENA) antibody, IgG	Negative
Smith (ENA) antibody, IgG	Negative
Scleroderma SCL-70 Ab	Negative
Anti-JO1 Ab	Negative
Serine protease 3 (PR-3) IgG	Positive – 68 (H) (normal 0–10 AU/mL)
Myeloperoxidase antibodies, IgG	Negative
Anticardiolipin Ab IgA, IgG, IgM	Negative
Beta-2 glycoprotein 1 IgG, IgM Ab	Negative

CMR performed before OSH (outside hospital) discharge showed a recovered LVEF of 57% and a nonischemic pattern. There was LGE with mid-myocardial linear enhancement in multiple areas of the left ventricle, including the basal and mid-inferoseptal wall, extending to the anteroseptal and inferior basal segments. The differential diagnosis included myocarditis, sarcoidosis, or genetic cardiomyopathy. Previously ordered chest X-ray and CT chest, along with a normal angiotensin-converting enzyme (ACE) level, did not support findings consistent with sarcoidosis. In summary, the newly positive Coxsackie B viral antibody titers, the reduced ejection fraction, improving from <20% to 57% with treatment, complicated by cardiogenic shock, the elevated inflammatory markers, and the CMR findings confirmed a diagnosis of Coxsackie B virus-induced fulminant myocarditis.

## Discussion

This case demonstrates the challenges associated with the diagnosis of viral myocarditis. While the history on presentation was suggestive of a viral infection, initial testing did not confirm the diagnosis. Interestingly, the PR3 antibody test was positive, but in isolation, it has been known to be positive in viral infections [4]. Respiratory viral panels are not perfectly sensitive and do not test for every respiratory virus that may affect the patient. Our initial Coxsackie B virus antibody panel was negative, which is often the case in the early stage of infection, given the time needed for the immune system to produce antibodies [5]. A positive test with elevated titers, confirming acute or recent infection, can occur up to two weeks after the initial symptoms. The decision for repeat testing when clinical suspicion remains high is appropriate [5]. In our case, repeat testing unveiled the final diagnosis. Making an accurate diagnosis is important, as treatment is targeted to the underlying etiology.

Among cases of viral myocarditis, human herpesvirus 6 (HHV-6) and parvovirus B19 were the most common culprits from the 1980s to the 2000s [1]. More recently, SARS-CoV-2 has emerged as a common cause, potentially skewing epidemiologic data on viral myocarditis [1]. Other common viral agents include HIV, HCV, and influenza A and B [1]. Coxsackie B virus is a rarer cause in adults but has a higher prevalence in children and adolescents. Coxsackie B affects cardiomyocytes through both direct viral cytotoxicity, by hijacking specific transmembrane channels, and activation of the innate and adaptive immune responses [6]. While in our case there were also elevated echovirus titers, Coxsackie B was considered the most likely etiology rather than a coinfection, given the limited cases of echovirus-induced myocarditis [7].

Treatment is dependent on the etiology and severity of the presentation. Mild cases primarily present with minimal symptoms, such as chest pain, and often self-resolve. Unlike pericarditis, treatment with NSAIDs (nonsteroidal anti-inflammatory drugs) is not recommended [6]. Severe cases involve one or more of the following: left ventricular systolic dysfunction, acute heart failure, ventricular arrhythmias, advanced atrioventricular conduction disturbances, or cardiogenic shock. Patients with complicated myocarditis and reduced left ventricular function benefit from heart failure GDMT [1].

In fulminant myocarditis, the role of advanced heart failure therapies depends on the severity and chronicity of myocardial injury. In patients with predominant left ventricular failure, an IABP or an axial-flow supporting device (e.g., Impella) may be indicated, whereas venoarterial extracorporeal membrane oxygenation (VA-ECMO) is ideal for cases with biventricular involvement or concomitant respiratory failure [2]. In patients with cardiogenic shock, in whom recovery is expected to be prolonged, implantation of assist devices as a bridge-to-transplant or recovery may be effective [1]. In cases where maximal medical therapy combined with mechanical circulatory support is insufficient, heart transplantation should be considered.

The role of immunosuppressants and immunomodulators (IVIG) is controversial in the treatment of acute myocarditis, and their use is not universally recommended. Trial data are limited, and most studies look at non-viral etiologies [6]. Few studies have noted benefits in overall improvement in LVEF in those treated with prednisone only and a combination of prednisone and azathioprine, while others, such as the Myocarditis Treatment Trial, have shown no significant difference in LVEF and mortality between immunosuppressants versus placebo [6]. Given the contradictory data, the European Society of Cardiology recommends the use of immunosuppressants in the treatment of viral-negative, eosinophilic, giant-cell, and autoimmune myocarditis [6]. The use of IVIG in viral myocarditis is investigational, with a limited number of studies exploring LVEF improvement and mortality benefit [6]. IVIG is clinically used in antibody-mediated autoimmune myocarditis. Antiviral therapies, including acyclovir, valacyclovir, and ganciclovir, are not well-tested and therefore are not used in the treatment of viral myocarditis [6]. As in our case, it must be acknowledged that without histological subtyping from endomyocardial biopsy, choosing appropriate treatment is further complicated, and empiric multimodal regimens may be necessary for clinical stability.

Viral myocarditis carries significant long-term mortality, with a 15% risk of cardiac mortality at 4.7 years according to a study of patients with biopsy-proven viral myocarditis (a population with likely more severe presentation on average) [8]. A CMR finding of LGE, as present on our patient’s CMR, yields a hazard ratio of 8.4 for all-cause mortality and 12.8 for cardiac mortality, independent of New York Heart Association (NYHA) heart failure classifications and primary LVEF [8]. This indicates that despite our patient’s remarkable LVEF recovery, the presence of LGE serves as a more significant prognostic factor for his long-term mortality. Additionally, primary NYHA Classes II-IV are strong predictors for incomplete recovery in subsequent follow-up, which does not necessarily correlate with our patient, who presented with NYHA Class II/III symptoms and ended up with complete LVEF recovery [8]. Regardless, these data highlight the importance of CMR and initial clinical presentation for risk stratification and long-term prognostication. More aggressive management with close monitoring for complications such as arrhythmias and multiorgan failure, and strict adherence to heart failure GDMT, is paramount to reducing long-term mortality.

## Conclusions

This case describes an uncommon presentation of fulminant myocarditis secondary to a viral infection, confirmed by clinical presentation, viral titers, and LGE distribution on CMR. It explores the challenging and broad management strategy employed in the absence of an endomyocardial biopsy, which fortunately resulted in a good outcome for this patient. Given the lack of histological subtyping, it is difficult to determine the true effect of controversial immunosuppressants and immunomodulators. However, this case highlights the importance of heart failure GDMT and hemodynamic support, including Impella devices and IABPs. The LGE distribution observed on CMR serves as a strong prognostic indicator for all-cause and cardiac mortality, warranting close follow-up care for the patient. Additionally, this case highlights the gaps in knowledge surrounding fulminant myocarditis management, particularly in the absence of an endomyocardial biopsy, and emphasizes the importance of integrating clinical judgment, including repeated diagnostics, into a multifaceted therapeutic approach.

## References

[1] Sozzi FB, Gherbesi E, Faggiano A (2022). Viral myocarditis: classification, diagnosis, and clinical implications. Front Cardiovasc Med.

[2] Pahuja M, Adegbala O, Mishra T (2019). Trends in the incidence of in-hospital mortality, cardiogenic shock, and utilization of mechanical circulatory support devices in myocarditis (analysis of National Inpatient Sample data, 2005-2014). J Card Fail.

[3] Montero S, Abrams D, Ammirati E (2022). Fulminant myocarditis in adults: a narrative review. J Geriatr Cardiol.

[4] Choi H, Park YB, Song J, Lee SW (2022). Unclassifiable repeated antineutrophil cytoplasmic antibody (ANCA) positivity in diseases other than ANCA-associated vasculitis. Z Rheumatol.

[5] Kordi R, Chang AJ, Hicar MD (2024). Seasonal testing, results, and effect of the pandemic on coxsackievirus serum studies. Microorganisms.

[6] Pollack A, Kontorovich AR, Fuster V, Dec GW (2015). Viral myocarditis--diagnosis, treatment options, and current controversies. Nat Rev Cardiol.

[7] Mirabel M, Callon D, Bruneval P (2020). Late-onset giant cell myocarditis due to enterovirus during treatment with immune checkpoint inhibitors. JACC CardioOncol.

[8] Grün S, Schumm J, Greulich S (2012). Long-term follow-up of biopsy-proven viral myocarditis: predictors of mortality and incomplete recovery. J Am Coll Cardiol.

